# Al_0.5_Nb_1.5_(PO_4_)_3_

**DOI:** 10.1107/S1600536811003886

**Published:** 2011-02-12

**Authors:** Dan Zhao, Peng Liang, Ling Su, Huan Chang, Shi Yan

**Affiliations:** a Department of Physics and Chemistry, Henan Polytechnic University, Jiaozuo, Henan 454000, People’s Republic of China

## Abstract

Single crystals of the title compound, aluminium niobium triphosphate, Al_0.5_Nb_1.5_(PO_4_)_3_, have been synthesized by a high-temperature reaction in a platinium crucible. The Al^III^ and Nb^V^ atoms occupy the same site on the 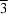 axis, with disorder in the ratio of 1:3. The fundamental building units of the title structure are isolated Al/NbO_6_ octa­hedra and PO_4_ tetra­hedra (. 2 symmetry), which are further inter­locked by corner-sharing O atoms, leading to a three-dimensional framework structure with infinite channels along the *a* axis.

## Related literature

For related structures, see: Aatiq & Bakri, (2007 ▶); Boilot *et al.* (1987 ▶); Chakir *et al.* (2006) ▶; Hong (1976 ▶); Masquelier *et al.* (2000 ▶); Trubach *et al.* (2004 ▶); Rodrigo *et al.* (1989 ▶); Zatovskii *et al.* (2006 ▶); Zhao *et al.* (2009 ▶). For compounds with the same structure type, see: Benmokhtar *et al.* (2007 ▶); Leclaire *et al.* (1989 ▶). For related structures, see: Brochu *et al.* (1997 ▶).

## Experimental

### 

#### Crystal data

Al_0.5_Nb_1.5_(PO_4_)_3_
*M* *_r_* = 437.76
Trigonal, 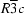

*a* = 8.5679 (6) Å
*c* = 21.898 (2) Å
*V* = 1392.14 (19) Å^3^
*Z* = 6
Mo *K*α radiation
μ = 2.51 mm^−1^
*T* = 293 K
0.15 × 0.05 × 0.05 mm

#### Data collection

Bruker SMART 1K CCD area-detector diffractometer
Absorption correction: multi-scan (*SADABS*; Bruker, 1997 ▶) *T*_min_ = 0.704, *T*_max_ = 0.885
2295 measured reflections
302 independent reflections
298 reflections with *I* > 2σ(*I*)
*R*_int_ = 0.029

#### Refinement

*R*[*F* ^2^ > 2σ(*F* ^2^)] = 0.027
*wR*(*F* ^2^) = 0.064
*S* = 1.39
302 reflections
27 parameters
Δρ_max_ = 0.45 e Å^−3^
Δρ_min_ = −0.39 e Å^−3^

### 

Data collection: *SMART* (Bruker, 1997 ▶); cell refinement: *SAINT* (Bruker, 1997 ▶); data reduction: *SAINT*; program(s) used to solve structure: *SHELXS97* (Sheldrick, 2008 ▶); program(s) used to refine structure: *SHELXL97* (Sheldrick, 2008 ▶); molecular graphics: *DIAMOND* (Brandenburg, 2004 ▶); software used to prepare material for publication: *SHELXTL* (Sheldrick, 2008 ▶).

## Supplementary Material

Crystal structure: contains datablocks I, global. DOI: 10.1107/S1600536811003886/fj2384sup1.cif

Structure factors: contains datablocks I. DOI: 10.1107/S1600536811003886/fj2384Isup2.hkl

Additional supplementary materials:  crystallographic information; 3D view; checkCIF report

## Figures and Tables

**Fig. 1.**
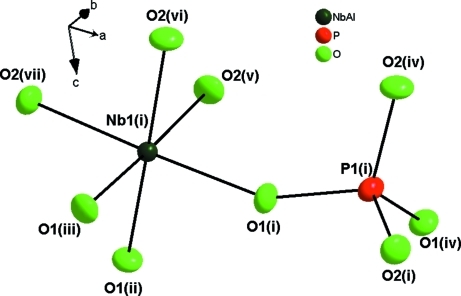
The expanded asymmetric unit of Al0.5Nb1.5(PO4)3 showing the coordination environments of the P and Al/Nb atoms. The displacement ellipsoids are drawn at the 50% probability level.[Symmetry codes: (i) x, y, z; (ii) -x + y, -x, z; (iii) -y, x-y, z; (iv) 0.66667 - x, 0.33333 - x + y, 0.83333 - z; (v) 0.66667 - y, 0.33333 - x, -0.16667 + z; (vi) -1/3 + x, 1/3 + x-y, -0.16667 + z; (vii) -0.33333 - x + y, -2/3 + y, -0.16667 + z.]

**Fig. 2.**
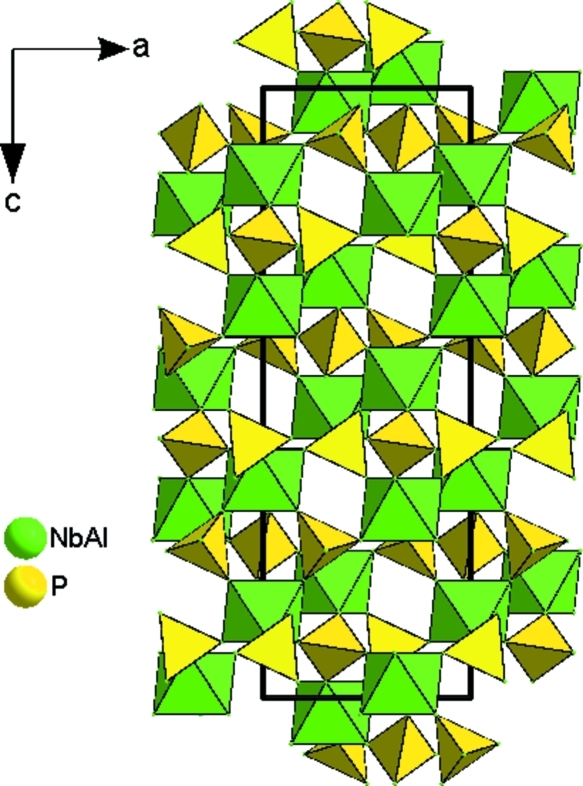
View of the crystal structure of Al0.5Nb1.5(PO4)3 along [010]. PO4 and Al/NbO6 units are given in the polyhedral representation.

## supplementary crystallographic information

### Comment

The mixed phosphates *AM*_2_(PO_4_)_3_ family (*A* = alkali metals; *M* = Ti, Zr, Ge, Sn) which usually belong to the NASICON (Na_3_Zr_2_Si_2_PO_12_: Boilot, *et al.*, 1987) or the NZP (NaZr_2_(PO_4_)_3_: Hong, 1976) structure-type have been extensively investigated for the low thermal expansion behavior of some members. The crystal structure that features a flexible three-dimensional framework of PO_4_ tetrahedra sharing comers with MO_6_ octahedra, is amenable to a wide variety of chemical substitutions at the various crystallographic positions, thus yielding a large number of closely related compounds, such as Na_3_MgZr(PO_4_)_3_ (Chakir, *et al.*, 2006), Na_3_Fe_2_(PO_4_)_3_ (Masquelier, *et al.*, 2000), NaFeNb(PO_4_)_3_ (Zatovskii, *et al.*, 2006), NaTi_2_(PO_4_)_3_ (Rodrigo, *et al.*, 1989) and NaGe_2_P_3_O12 (Zhao *et al.*, 2009). The three-dimensional network consisting of PO_4_ and MO_6_ octahedra delimit two different types of channels in which the *A* atoms are usually located to compensate the negative charges. It is reported that the *A* atoms can completely empty in some areas, such as Fe_0.5_Nb_1.5_(PO_4_)_3_ (Trubach, *et al.*, 2004) and Fe_0.5_Sb_1.5_(PO_4_)_3_ (Aatiq & Bakri, 2007), Nb_2_(PO_4_)_3_(Leclaire, *et al.*,1989) and Fe_0.5_Ti_2_(PO_4_)_3_(Benmokhtar, *et al.*, 2007), *etc*. In order to inrich this type of compounds, we synthesis the compound Al_0.5_Nb_1.5_(PO_4_)_3_ by a high-temperature reaction and determine the crystal structure from single-crystal X-ray diffraction analysis.

As shown in Fig. 1, the asymmetric unit of Al_0.5_Nb_1.5_(PO_4_)_3_ contains a single P and Al/Nb atoms. The P atom is four coordinated by four oxygen atoms, forming isolated PO_4_ tetrahedron. Al and Nb atoms are in mixed occupancy disorder locating at the 3 axes with the moral ratio of 1: 3, being coordinated by six oxygen atoms to form Al/NbO_6_ octahedra. Al/NbO_6_ octahedra and PO_4_ tetrahedra are further interconnected *via* corner-sharing O atoms to form the three-dimensional framework of Al_0.5_Nb_1.5_(PO_4_)_3_, as shown in Fig. 2. The Al/Nb—O bonds have two groups of different distances, that is, 1.913 (3) and 1.949 (3) Å. The PO_4_ tetrahedra are regular with two groups of P–O bond distances of 1.521 (3) and 1.529 (3) Å, and O–P–O bond angles weak dispersion from 107.91 (16) to 111.3 (2)*^o^*, which is about the ideal value of 109.48°. On the other hand, this structure can be viewed as a NZP structure, in which the Na atom sites empty and the Zr atoms site are replaced by Al and Nb atoms in disordered manner on the principle of aliovalent pair combination Zr^4+^→ 0.25 A l^3+^ + 0.73 N b^5+^.

### Experimental

The finely ground reagents K_2_CO_3_, Al_2_O_3_, Nb_2_O_5_ and NH_4_H_2_PO_4_ were mixed in the molar ratio K: Al: Nb: P = 1: 3: 10: 20, were placed in a Pt crucible, and heated at 573 K for 4 h. The mixture was then re-ground and heated at 1473 K for 20 h, then cooled to 973 K at a rate of 3 K h^-1^, and finally quenched to room temperature. A few colorless crystals of the title compound with prismatic shape were obtained.

### Refinement

The structure contains substitutional disorder in which Al1 and Nb1 occupy the same position. The atomic positional and anisotropic displacement parameters of Al1 and Nb1 atoms were constrained to be identical by using EADP and EXYZ constraint instructions (*SHELXL97*; Sheldrick, 2008). The ratio of Al1 and Nb1 was fixed to 1: 3 to achieve charge balance.

### Crystal data

**Table:**

Al_0.5_Nb_1.5_(PO_4_)_3_	*D*_x_ = 3.133 Mg m^−^^3^
*M**_r_* = 437.76	Mo *K*α radiation, λ = 0.71073 Å
Trigonal, *R*3*c*	Cell parameters from 247 reflections
Hall symbol: -R 3 2"c	θ = 2.6–25.0°
*a* = 8.5679 (6) Å	µ = 2.51 mm^−^^1^
*c* = 21.898 (2) Å	*T* = 293 K
*V* = 1392.14 (19) Å^3^	Prism, colourless
*Z* = 6	0.15 × 0.05 × 0.05 mm
*F*(000) = 1254	

### Data collection

**Table:**

Bruker SMART 1K CCD area-detector diffractometer	302 independent reflections
Radiation source: fine-focus sealed tube	298 reflections with *I* > 2σ(*I*)
graphite	*R*_int_ = 0.029
ω scans	θ_max_ = 25.7°, θ_min_ = 3.3°
Absorption correction: multi-scan (*SADABS*; Bruker, 1997)	*h* = −7→10
*T*_min_ = 0.704, *T*_max_ = 0.885	*k* = −10→8
2295 measured reflections	*l* = −26→21

### Refinement

**Table:**

Refinement on *F*^2^	0 restraints
Least-squares matrix: full	Primary atom site location: structure-invariant direct methods
*R*[*F*^2^ > 2σ(*F*^2^)] = 0.027	Secondary atom site location: difference Fourier map
*wR*(*F*^2^) = 0.064	*w* = 1/[σ^2^(*F*_o_^2^) + (0.0163*P*)^2^ + 17.3988*P*] where *P* = (*F*_o_^2^ + 2*F*_c_^2^)/3
*S* = 1.39	(Δ/σ)_max_ < 0.001
302 reflections	Δρ_max_ = 0.45 e Å^−^^3^
27 parameters	Δρ_min_ = −0.39 e Å^−^^3^

### Special details

**Table:**

Geometry. All e.s.d.'s (except the e.s.d. in the dihedral angle between two l.s. planes) are estimated using the full covariance matrix. The cell e.s.d.'s are taken into account individually in the estimation of e.s.d.'s in distances, angles and torsion angles; correlations between e.s.d.'s in cell parameters are only used when they are defined by crystal symmetry. An approximate (isotropic) treatment of cell e.s.d.'s is used for estimating e.s.d.'s involving l.s. planes.
Refinement. Refinement of *F*^2^ against ALL reflections. The weighted *R*-factor *wR* and goodness of fit *S* are based on *F*^2^, conventional *R*-factors *R* are based on *F*, with *F* set to zero for negative *F*^2^. The threshold expression of *F*^2^ > σ(*F*^2^) is used only for calculating *R*-factors(gt) *etc*. and is not relevant to the choice of reflections for refinement. *R*-factors based on *F*^2^ are statistically about twice as large as those based on *F*, and *R*- factors based on ALL data will be even larger.

### Fractional atomic coordinates and isotropic or equivalent isotropic displacement parameters (Å^2^)

**Table:**

	*x*	*y*	*z*	*U*_iso_*/*U*_eq_	Occ. (<1)
Nb1	0.0000	0.0000	0.35896 (3)	0.0091 (2)	0.75
Al1	0.0000	0.0000	0.35896 (3)	0.0091 (2)	0.25
P1	0.3333	0.38482 (17)	0.4167	0.0143 (4)	
O1	0.1675 (4)	0.1984 (4)	0.40796 (12)	0.0173 (6)	
O2	0.3025 (4)	0.4696 (4)	0.47305 (12)	0.0194 (7)	

### Atomic displacement parameters (Å^2^)

**Table:**

	*U*^11^	*U*^22^	*U*^33^	*U*^12^	*U*^13^	*U*^23^
Nb1	0.0092 (3)	0.0092 (3)	0.0090 (4)	0.00460 (14)	0.000	0.000
Al1	0.0092 (3)	0.0092 (3)	0.0090 (4)	0.00460 (14)	0.000	0.000
P1	0.0179 (8)	0.0126 (5)	0.0141 (7)	0.0089 (4)	−0.0043 (6)	−0.0022 (3)
O1	0.0172 (15)	0.0132 (14)	0.0183 (14)	0.0053 (13)	−0.0039 (12)	−0.0051 (11)
O2	0.0253 (16)	0.0164 (15)	0.0162 (14)	0.0102 (14)	−0.0008 (12)	−0.0052 (11)

### Geometric parameters (Å, °)

**Table:**

Nb1—O1	1.913 (3)	P1—O2	1.521 (3)
Nb1—O1^i^	1.913 (3)	P1—O2^vi^	1.521 (3)
Nb1—O1^ii^	1.913 (3)	P1—O1^vi^	1.529 (3)
Nb1—O2^iii^	1.949 (3)	P1—O1	1.529 (3)
Nb1—O2^iv^	1.949 (3)	O2—Al1^vii^	1.949 (3)
Nb1—O2^v^	1.949 (3)	O2—Nb1^vii^	1.949 (3)
			
O1—Nb1—O1^i^	91.63 (12)	O1^ii^—Nb1—O2^v^	89.81 (12)
O1—Nb1—O1^ii^	91.63 (12)	O2^iii^—Nb1—O2^v^	88.66 (12)
O1^i^—Nb1—O1^ii^	91.63 (12)	O2^iv^—Nb1—O2^v^	88.66 (12)
O1—Nb1—O2^iii^	89.81 (12)	O2—P1—O2^vi^	111.3 (2)
O1^i^—Nb1—O2^iii^	89.86 (12)	O2—P1—O1^vi^	110.32 (15)
O1^ii^—Nb1—O2^iii^	177.90 (12)	O2^vi^—P1—O1^vi^	107.91 (16)
O1—Nb1—O2^iv^	177.90 (12)	O2—P1—O1	107.91 (16)
O1^i^—Nb1—O2^iv^	89.81 (12)	O2^vi^—P1—O1	110.32 (15)
O1^ii^—Nb1—O2^iv^	89.86 (12)	O1^vi^—P1—O1	109.1 (2)
O2^iii^—Nb1—O2^iv^	88.66 (12)	P1—O1—Nb1	152.96 (18)
O1—Nb1—O2^v^	89.86 (12)	P1—O2—Al1^vii^	155.8 (2)
O1^i^—Nb1—O2^v^	177.90 (12)	P1—O2—Nb1^vii^	155.8 (2)

Symmetry codes: (i) −*x*+*y*, −*x*, *z*; (ii) −*y*, *x*−*y*, *z*; (iii) −*y*+2/3, −*x*+1/3, *z*−1/6; (iv) −*x*+*y*−1/3, *y*−2/3, *z*−1/6; (v) *x*−1/3, *x*−*y*+1/3, *z*−1/6; (vi) −*x*+2/3, −*x*+*y*+1/3, −*z*+5/6; (vii) −*x*+*y*+1/3, *y*+2/3, *z*+1/6.

## References

[bb1] Aatiq, A. & Bakri, R. (2007). *Powder Diffr.* **22**, 47–54.

[bb2] Benmokhtar, S., El Jazouli, A., Aatiq, A., Chaminade, J. P., Gravereau, P., Wattiaux, A., Fournes, L. & Grenier, J. C. (2007). *J. Solid State Chem.* **180**, 2004–2012.

[bb3] Boilot, J. P., Collin, G. & Colomban, Ph. (1987). *Mater. Res. Bull.* **22**, 669–676.

[bb4] Brandenburg, K. (2004). *DIAMOND.* Crystal Impact GbR, Bonn, Germany.

[bb5] Brochu, R., Louer, M., Alami, M., Alqaraoui, M. & Louer, D. (1997). *Mater. Res. Bull.* **32**, 113–122.

[bb6] Bruker (1997). *SMART*, *SAINT* and *SADABS.* Bruker AXS Inc., Madison, Wisconsin, USA.

[bb15] Chakir, M., El Jazouli, A. & de Waal, D. (2006). *J. Solid State Chem.* **179**, 1883–1891.

[bb7] Hong, H. Y. P. (1976). *Mater. Res. Bull.* **11**, 173–182.

[bb8] Leclaire, A., Borel, M.-M., Grandin, A. & Raveau, B. (1989). *Acta Cryst.* C**45**, 699–701.

[bb9] Masquelier, C., Wurm, C., Rodriguez-Carvajal, J., Gaubicher, J. & Nazar, L. (2000). *Chem. Mater.* **12**, 525–532.

[bb10] Rodrigo, J. L., Carrasco, M. P. & Alamo, J. (1989). *Mater. Res. Bull.* **24**, 611–618.

[bb11] Sheldrick, G. M. (2008). *Acta Cryst.* A**64**, 112–122. 10.1107/S0108767307043930 18156677

[bb12] Trubach, I. G., Orlova, A. I., Beskrovnyi, A. I., Koryttseva, A. K., Zharinova, M. V., Kurazhkovskaya, V. S. & Lipatova, E. V. (2004). *Crystallogr. Rep.* **49**, 396–400.

[bb13] Zatovskii, I. V., Ushchapovskaya, T. I., Slobodyanik, N. S. & Ogorodnik, I. V. (2006). *Zh. Neorg. Khim.* **51**, 41–46.

[bb14] Zhao, D., Xie, Z., Hu, J. M., Zhang, H., Zhang, W. L., Yang, S. L. & Cheng, W. D. (2009). *J. Mol. Struct.* **922**, 127–134.

